# A Precise Multi-Exposure Image Fusion Method Based on Low-level Features

**DOI:** 10.3390/s20061597

**Published:** 2020-03-13

**Authors:** Guanqiu Qi, Liang Chang, Yaqin Luo, Yinong Chen, Zhiqin Zhu, Shujuan Wang

**Affiliations:** 1 Computer Information Systems Department, State University of New York at Buffalo State, Buffalo, NY 14222, USA; qig@buffalostate.edu; 2 College of Automation, Chongqing University of Posts and Telecommunications, Chongqing 400065, China; 1452380548cl@gmail.com (L.C.); luozi233@outlook.com (Y.L.); zhuzq@cqupt.edu.cn (Z.Z.); 3 School of Computing, Informatics, and Decision Systems Engineering, Arizona State University, Tempe, AZ 85287, USA; yinong@asu.edu; 4 Faculty of Information Engineering and Automation, Kunming University of Science and Technology, Kunming 650500, Yunnan, China

**Keywords:** multi-exposure image fusion, high-dynamic-range imaging, ghost removal, image fusion, a priori exposure quality

## Abstract

Multi exposure image fusion (MEF) provides a concise way to generate high-dynamic-range (HDR) images. Although the precise fusion can be achieved by existing MEF methods in different static scenes, the corresponding performance of ghost removal varies in different dynamic scenes. This paper proposes a precise MEF method based on feature patches (FPM) to improve the robustness of ghost removal in a dynamic scene. A reference image is selected by a priori exposure quality first and then used in the structure consistency test to solve the image ghosting issues existing in the dynamic scene MEF. Source images are decomposed into spatial-domain structures by a guided filter. Both the base and detail layer of the decomposed images are fused to achieve the MEF. The structure decomposition of the image patch and the appropriate exposure evaluation are integrated into the proposed solution. Both global and local exposures are optimized to improve the fusion performance. Compared with six existing MEF methods, the proposed FPM not only improves the robustness of ghost removal in a dynamic scene, but also performs well in color saturation, image sharpness, and local detail processing.

## 1. Introduction

Existing imaging devices cannot capture all the details in a scene by a one-time exposure. As the main reason, there is a mismatch of the dynamic range in response to a real scene between an imaging device and human eyes. This seriously affects the visual effects of imaging and the retention of key information [1,2]. Generally, human eyes have a wider dynamic range than an imaging device. For both color and brightness, the image captured by an imaging device is different from the one observed by human eyes in a real scene, such as an image captured in a night scene. Therefore, HDR imaging techniques are introduced to solve the above-mentioned mismatch issues.

As a type of high-dynamic-range (HDR) imaging techniques, multi-exposure image fusion (MEF) related techniques can extract the comprehensive image information from different exposure images and combine them into an image. MEF has drawn wide attention since the first publication in 1980 [3]. In the early stage of MEF, Debevec used the camera-response curves to obtain HDR images. As the solid foundation for the later research, DiCarlo divided an HDR image into the corresponding units first [4] and then mapped the obtained units to 8 bit integer numbers in the range [0,255] [5]. Most of the existing MEF algorithms are based on the spatial-domain methods [6,7,8,9]. According to certain rules, the spatial-domain based fusion methods directly merge multiple different exposure source images at the pixel level. Due to the small displacements caused by device shaking or object motion, most of the existing fusion methods are prone to ghosting in practical applications. Therefore, image registration is required, when MEF is applied to a dynamic scene. Compared with the object motion, device motion can be handled by fixed equipment or registration techniques. Therefore, existing algorithms mainly focus on the duplication removal of object motion, which is categorized into two main categories: reference and non-reference based algorithms. According to existing fusion algorithms without reference images, non-reference based algorithms depend on the statistical distribution of pixels [10,11,12,13,14,15,16]. They assume a moving object only appears in a relatively small number of input images at a certain position. Therefore, multiple images are needed as input. Based on the statistical distribution of pixel points, motion estimation is performed by using a median threshold bitmap and a proportional relationship between image luminance and exposure time. The input image is selected from reference images as a motion reference by a ghost removal method [15,17]. The motion estimation uses the intensity mapping function (IMF) [17] and analyzes the structure consistency among different multi-exposure source images [18], which can retain the moving objects contained in the reference images.

The proposed solution uses filters to decompose source images into image patches for further processing, which is different from the per-pixel MEF method commonly used in existing solutions. Specifically, it adds the a priori exposure quality of input images first, which selects an image with the best exposure quality from a set of input images as a reference for other images. If the a priori exposure quality is not used, an image with poor exposure quality may be selected as a reference image to reduce the exposure quality of the fused image. Then, it uses IMF [17] to solve the ghosting issues of MEF in a dynamic scene. Next, it uses a guided filter to decompose each input source image into the base layer and detail layer, which is a fast two-scale image decomposition method. Specifically, an image patch of the base layer is decomposed into three conceptually independent components: signal strength, signal structure, and mean intensity. Each component is processed individually based on patch strength, exposedness, and structural consistency. The color information is naturally used by processing the RGB color channels of an image patch jointly. Moreover, the structural consistency of multi-exposure patches from the base layer can be easily checked by the direction information of the signal structure component. Therefore, a high-quality fused image with little ghosting artifacts can be obtained from the base layer that contains a large number of intensity variations. After improving the exposure quality of the base layer, it uses the luminance components of each input source image to extract the exposedness features, which are mapped to the mixed weights of both the base and detail layers by an exposedness function. The Gaussian function is constructed by using the image luminance information to evaluate the exposure quality of an image. The fusion performance is improved by the optimization of both global and local exposures to achieve the accurate and fast fusion of multi-exposure images. Finally, it combines both the base and detail layers to obtain the fused image. The proposed image fusion framework has three main contributions:

1. An image registration algorithm based on the a priori exposure quality is proposed. It minimizes the local exposure distortion of the fused image caused by the improper selection of the reference image to improve the robustness of MEF in a dynamic scene. Additionally, both the structure consistency test and connectivity test are introduced to identify the ghost regions in the ghost removal process. The structure consistency detection can effectively avoid a large amount of display motion estimations.
2. An MEF framework based on the low-level image features is proposed. It integrates the spatial-domain scale decomposition, image patch structure decomposition, and the moderate exposure evaluation that optimize both global and local image exposure qualities to improve the visual image quality. In addition, the low-level features such as image brightness, contrast, and intensity are used to improve the fusion efficiency, which preserve more detailed information of a scene. Therefore, it achieves the precise fusion of multi-exposure images.
3. The proposed MEF framework can not only be used in a static scene, but also in a dynamic scene. Comparing with existing MEF solutions, the proposed MEF framework improves the robustness of ghost removal in a dynamic scene and performs well in image color saturation, sharpness, and local detail processing. The performance of the proposed framework is confirmed in both subjective and objective evaluations.

The remaining sections of this paper are structured as follows: Section 2 discusses existing solutions of both image fusion in a static scene and ghost removal in a dynamic scene; Section 3 proposes an accurate and fast fusion framework based on the low-level image features; Section 4 compares and analyzes the experiment results; and Section 5 concludes this paper.

## 2. Related Work

The existing MEF solutions mainly face two difficulties, such as the precise fusion in a static scene and the ghost removal in a dynamic scene.

### 2.1. MEF Algorithms in A Static Scene

Existing MEF solutions are mainly applicable to various static scenes, which can be categorized into transform- and spatial-domain solutions. Transform-domain based image fusion methods transform several images with different exposures to transformation coefficients in different ways first. Then, the corresponding transform coefficients are selected from a set of images with different exposures taken at the same spatial location to form a new set of transform coefficients, which are inversely transformed to obtain the fused image [19,20]. Mertens proposed a multi-resolution method based on the Laplacian pyramid [21]. Shen proposed an MEF method based on the hybrid weight and the improved Laplacian pyramid [22]. The improved Laplacian pyramid can enhance image details to ensure the fused image has rich colors. Li established an MEF model based on the median and recursive filtering [9]. The fast generation of an HDR image is realized by the comprehensive evaluation of image contrast, color, and brightness. MEF algorithms based on the transform-domain involve Laplacian pyramid transform, which may cause the detail loss of the fused image [23,24].

The spatial-domain based image fusion methods process the input images at the pixel level first and then combine multiple images with difference exposures in the spatial domain following certain rules to obtain a fused image [23,25]. Compared with transform-domain based image fusion methods, spatial-domain based methods save more details of source images, which are suitable for MEF. Kinoshita proposed an MEF method based on the exposure compensation [26]. Prabhakar proposed an algorithm to generate a ghost-free HDR image by fusing a set of multi-exposure images in the gradient domain [27]. Liu introduced a quality measurement method to scale-invariant feature transform (SIFT) based MEF [28]. Local details can be extracted from source images using a dense SIFT descriptor called “activity level” measurement first. Then, they are used to remove the ghost artifacts when the captured scene and moving objects are dynamic. The pixel based fusion methods require blending weights [22], median filtering [9], and gradient-domain least squares [29] to process the weight map, which is used to reduce the fusion artifacts [30,31]. Therefore, Ma proposed a fusion algorithm based on the decomposition of the patch structure to generate the noise-free weight maps and vivid high-quality fused images [30]. This method does not require the subsequent processing steps to improve the visual quality or reduce the spatial artifacts. However, parts of the fused result are overexposed, which causes the loss of details and affects the fusion performance [31]. Then, according to the patch structure, the similarity index of the color structure is introduced to achieve the fusion of MEF [31]. Although this method optimizes the quality of local exposure, the patch size is fixed, which causes the loss of the detailed structure and texture information in the fused image. According to the entropy of image texture, Li implemented the adaptive size selection of image patches [24,32]. This method solves the detail loss caused by the fixed size of the image block. However, it involves the entropy calculation of the image texture and the iterative optimization process. Therefore, the fusion process is long. In order to achieve rapid MEF, Nejati used the guided filter to scale source images first and then obtained the base and detail layers of source images, respectively [18]. This method reduces the fusion time by using the scale decomposition and the global exposure optimization, but the lack of local exposure optimization may cause the loss of local fine details. In addition, this method only works for MEF in a static scene, which cannot effectively remove the ghosting effects. Qin used a low-pass anti-aliasing filter to capture the signals by sampling at a relatively low rate, which reduced the burden on the analog hardware [33]. Liu applied different guided filtering methods to the source images decomposed by complex shearlet [34]. Two-scale and larger sum-modified-Laplacian guided filtering fusion rules were used to process low- and high-frequency coefficients, respectively. After the processing of guided filters, the information of source images was preserved well, and the spatial continuity of fused image was improved. Ma used a guided filter to upsample the weight maps jointly in the proposed fast multi-exposure image fusion solution [35]. Therefore, the perceptually calibrated MEF structural similarity (MEF-SSIM) index was optimized to do the related training over a database of training sequences at full resolution.

### 2.2. Ghost Removal Algorithms in A Dynamic Scene

Ghost effects may occur in the motion presence of a device and/or objects during the MEF process. In order to reduce the ghost effects, many ghost removal methods have been proposed and applied to dynamic scenes. For non-reference images, Wang proposed a ghost-free high-dynamic-range imaging (HDRI) algorithm based on the visual saliency [10]. Zhang used the consistency of gradient direction to determine whether an object had a motion [16]. Jacobs proposed a method to generate HDR images automatically from low-dynamic-range (LDR) images [3]. According to the analysis, the fused result was obtained by the weighted sum of multi-exposure input images [12,36]. Therefore, the traditional MEF methods were affected by the ghosting effects, when any object was moving or the hand was trembling. The weights of those pixels that caused the ghosting effects were eliminated, and the weights were reduced when the correlation between the exposed image and the reference image was not high. When any moving object occupied different areas in each source image, the non-reference image based ghost removal algorithms require multiple input images from the same scene [14]. Based on the reference image, Hu proposed an iterative method to use color mapping functions and intensity histograms [14]. This method corrected the pixel points that did not match at the same position between the input image and the reference image. Sen used the minimization formula of dynamic block energy to achieve the multi-exposure image registration in a dynamic scene [15]. This method could handle the various motions of both shaky camera and objects. However, it involved an iterative process, which caused the high computation cost. The MEF algorithm proposed by Li used IMF and the bidirectional normalization to detect the inconsistent pixel points and also applied the two-wheel hybrid correction method to the ghost removal [17]. In order to reduce the motion estimation cost, Ma generated a consistency map of the image structure by calculating the inner product of both multi-exposure image and reference image structure vectors [30]. This method used the spatial directivity of each image-block structure vector to detect the motion consistency and did not require an iterative optimization process.

Existing MEF algorithms have two main difficulties: the fusion in a static scene and the ghost removal in a dynamic scene. Since most of existing algorithms are applicable to static scenes, they lack the robustness to dynamic scenes. The fusion is achieved by the optimization of global or local exposure quality individually, so the corresponding visual effects of the fused result are affected. The local overexposure or underexposure caused by only using the global optimization appears in the fused image. Similarly, the overall fusion performance is lowered by only using the local optimization. Additionally, the saturated or heavily underexposed pixels of a reference image are not typically matchable during the ghost removal process in a dynamic scene, because these pixels may be detected as the outliers [14]. Due to the lack of a priori exposure quality, the loss of local details may occur in the fused image. This paper integrates the a priori exposure quality and the structure consistency test to improve the robustness of MEF. Meanwhile, the global and local exposure quality are optimized by the evaluation of exposure quality and the decomposition of the image patch structure.

## 3. Multi-exposure Image Registration Fusion Method

**Figure 1:**
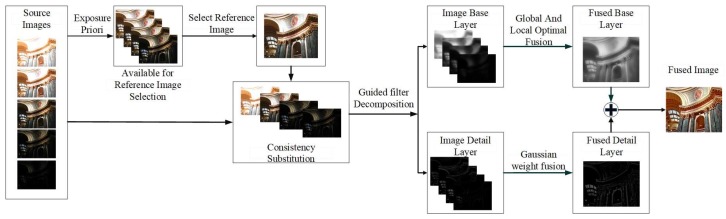
The proposed multi-exposure image fusion framework.

As shown in Figure 1, this paper proposes a novel MEF framework. Based on the analysis of structure consistency, image registration is applied to the ghost removal during the MEF process in a dynamic scene. Source images are decomposed in the spatial-domain first, and then, both the base and detail layer of the decomposed images are fused to achieve the MEF. With the structure decomposition of an image block and the appropriate evaluation of exposure, the fusion performance is improved by the optimization of both global and local exposure. The steps of the proposed algorithm are shown as follows.

### 3.1. Dynamic Scene Registration

#### 3.1.1. Reference Image Selection

This sets two thresholds α and β to specify the range in which the pixel value can be determined as overexposed/underexposed. The ratio of the number of pixels in the range to the number of pixels in the image is used to evaluate the image exposure quality. α as a scaling factor is used to work with $B_{t}$, which denotes the maximum value among all the pixels. $B_{t}$ represents an image pixel. The range and number of underexposed and overexposed pixels in source images are obtained by calculating α with $B_{t}$. The function of β is set as a scaling factor for the number of pixels in source images by calculating $s_{1}*s_{2}*\beta$, where $s_{1}$ and $s_{2}$ represent the image width and height, respectively. It determines whether a source image is underexposed or overexposed by comparing the obtained result as follows.

(1) $$max\left(t_{k},f_{k}\right)>s_{1}*s_{2}*\beta$$

where $t_{k}$ is the vector formed by the sum of all the pixels in $\left(B_{t}-B_{t}*\alpha ,B_{t}\right)$ of the $k^{\mathrm{th}}$ source image. $f_{k}$ is the vector similar to $t_{k}$, which is formed by the sum of all the pixels in $\left(0,B_{t}*\alpha \right)$. When the $k^{\mathrm{th}}$ source image satisfies Equation (1), it is not included in the selection of a reference image. It continues using Equation (1) to detect the exposure quality of the ${\left(k-1\right)}^{\mathrm{th}}$ source image. The proposed solution only selects one image as the reference image for the whole group of images. The selected reference image is the only image in source images that does not satisfy Equation (1). If there is more than one image that does not satisfy Equation (1), one of them is selected as the reference image randomly. If Equation (1) is satisfied by all source images, when *k* is an odd number, the ${\frac{k+1}{2}}^{\mathrm{th}}$ source image is selected as the reference image. Otherwise, the ${\frac{k}{2}}^{\mathrm{th}}$ source image is selected. Anyway, only one reference image is selected in this case.

**Figure 2:**
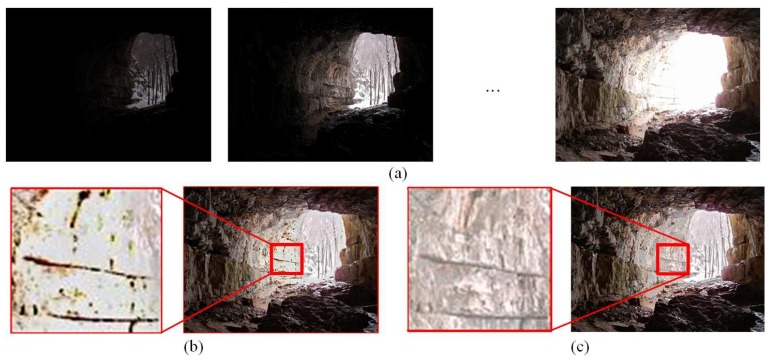
(**a**) Source images, (**b**) an image obtained by the reference image without a priori processing, and (**c**) an image obtained by the reference image with a priori processing.

When the selection of a reference image is completed, a description map is generated by a priori exposure quality and connectivity tests. The selection of underexposure or overexposure pixels can be avoided in the correction of inconsistent pixels. As shown in Figure 2, the application of a priori exposure quality can effectively improve the visual quality of the fused result.

#### 3.1.2. Intensity Map Replacement

Based on the reference image, it implements image registration by performing the scale-invariant feature transform (SIFT) matching. The analysis of structure consistency is used to detect the inconsistent areas by the reference image. Therefore, the inconsistent motions in the remaining images can be identified. Specifically, it uses a moving window with a fixed stride to extract image blocks from source images to get a set of $\{g_{k}\}=\{g_{k}\left|1\leq k\leq K\right\}$. In the image fusion process, the obtained set can provide the local image exposure features. $g_{k}$ is the column vector dimension. It can fully use the perceptually meaningful information scattered across different exposures in the same spatial location. Then, it calculates the inner product between the reference signal structure $S_{r}$ and the signal structure $S_{k}$ of another source image.

(2) $$\rho_{k}=S_{r}^{T}S_{k}=\frac{{\left(g_{r}-\mu_{r}\right)}^{T}\left(g_{k}-\mu_{k}\right)+d}{\left|\right|g_{r}-\mu_{r}\left|\right|\cdot \left|\right|g_{k}-\mu_{k}\left|\right|+d}$$

where ||·|| represents the norm of vector $l^{2}$, $s_{k}$ as the structure vector equals $\frac{g_{k}-\mu_{g_{k}}}{\left|\right|g_{k}-\mu_{g_{k}}\left|\right|}$, and $\mu_{g_{k}}$ is the average value of an image block. $\rho_{k}$ lies in $\left[-1,1\right]$. The larger $\rho_{k}$ means the higher consistency between $S_{r}$ and $S_{k}$. The constant *d* is used to ensure the robustness of the structure consistency to sensor noises. In order to detect as many inconsistent image blocks as possible, a threshold $T_{s}$ is introduced to binarize $\rho_{k}$, as shown in Equation (3).

(3) $${\tilde{C}}_{k}=\left\{\begin{array}{c} 1\;if\;\rho_{k}\geq T_{s}\\ 0\;if\;\rho_{k}\leq T \end{array}\right.$$

where ${\tilde{C}}_{k}$ is a structure consistency map. According to the structure consistency map of image blocks, the motion-inconsistency pixels are reliably identified in the entire dynamic scene. It adds another constraint in the ghost removal algorithm to achieve the mapping between the luminance values of any two exposures by Equation (3). Then, it minimizes the ghost areas by using IMF.

It creates k−1 latent images by mapping the intensity values of the reference image to the remaining k−1 exposures first, then calculates the absolute mean-intensity difference between the co-located patches in the $k^{\mathrm{th}}$ exposure and the corresponding latent image. The different thresholds are shown in Equation (4).

(4) $${\tilde{C}}_{k}\overset{⌢}{=}\{\begin{array}{c} 1\;if\;|l_{k}-{l^{\prime }}_{k}|<T_{m}\\ 0\;if\;|l_{k}-{l^{\prime }}_{k}|\geq T_{m} \end{array}$$

where $l_{k}^{\prime }$ is the mean intensity of the co-located patch in the $k^{\mathrm{th}}$ latent image created by the reference image and $T_{m}$ is a pre-defined threshold. The final structure consistency measure is defined by Equation (5).

(5) $$C_{k}={\bar{C}}_{k}\cdot {\tilde{C}}_{k}$$

where $C_{k}$ is used to select the corresponding image blocks in the latent image to compensate the ghost areas. Multi-exposure images of a dynamic scene are converted into a static scene image by using the ghost detection algorithm to avoid a large number of estimation calculations in the explicit motion. In addition, the average intensity of moving objects in the reference image can be optimally adjusted to be more suitable to the field environment. Finally, the high-quality fused result can be obtained.

### 3.2. A Precise Multi-Exposure Image Fusion

#### 3.2.1. Image Space-Domain Decomposition by the Guided Filter

The input images $\left\{M_{k}|1\leq k\leq K\right\}$ are decomposed into two-scale representations to obtain a smooth base layer with the large-scale intensity variations and a detail layer with the small-scale details. The weighted sum of RGB color channels is first used to calculate the luminance component $L_{k}$ corresponding to source images, and then, the guided filter is used as an effective edge-preservation smoothing filter [7] to obtain the base layer of source images as follows.

(6) $$B_{k}=G_{r,\delta }\left(L_{k},L_{k}\right)$$

where $G_{r,\delta }\left(P,Q\right)$ denotes the guided filtering operator, *r* is the filter radius, and δ controls the blurring degree. *P* and *Q* indicate the input image and guidance image, respectively. After obtaining the base layer of an image set, it is easy to obtain the corresponding detail layer $D_{k}$ of each image by using Equation (7).

(7) $$D_{k}=M_{k}-B_{k}$$

#### 3.2.2. Fusion Based on Global and Local Exposure Optimization

The quality optimization of global and local exposure achieved by the structure decomposition and exposure quality evaluation is used to optimize the fusion of the image base layer $B_{k}$. Similarly, it uses a fixed-size moving window to extract image blocks $\left\{b_{k}\right\}=\left\{b_{k}|1\leq k\leq K\right\}$ from the detail layer and then optimizes the partial exposure quality of image blocks. The structure decomposition algorithm of the image patch is used to obtain three independent parts, such as signal strength $c_{k}$, signal structure $s_{k}$, and mean intensity $l_{k}$ [30]. The desired signal strength of the fused image patch is determined by the highest signal strength of all the source image patches by Equation (8).

(8) $$\hat{c}=\max_{\left\{1\leq k\leq K\right\}}c_{k}=\max_{\left\{1\leq k\leq K\right\}}\left|\right|{\tilde{b}}_{k}\left|\right|$$

Different from the signal strength, the desired structure of the fused image patch is expected to represent the structures of all the source image patches. A simple implementation of this relationship represents the unit-length structure vector $s_{k}$ by Equation (9).

(9) $$\tilde{s}=\frac{\sum_{k=1}^{K}S({\tilde{b}}_{k})s_{k}}{\sum_{k=1}^{K}S({\tilde{b}}_{k})}\;and\;\hat{s}=\frac{\bar{s}}{\left|\right|\bar{s}\left|\right|}$$

where S(·) is a weighting function as defined by Equation (10) that determines the contribution of each source image patch in the fused image.

(10) $$S\left({\tilde{b}}_{k}\right)={\left|\right|{\tilde{b}}_{k}\left|\right|}^{p}$$

where p≥0 is an exponential parameter. Equation (10) employs a power weighting function. Since *p* has various values, a set of weighting functions with different physical meanings can be derived by the general formula shown as Equation (10). When the value of *p* gets larger, more emphasis is put on the image patch with relatively higher strength.

With regard to the mean intensity of the local patch, it takes a similar form of Equation (9) to generate Equation (11).

(11) $$\tilde{l}=\frac{\sum_{k=1}^{K}L(\mu_{k},l_{k})l_{k}}{\sum_{k=1}^{K}L(\mu_{k},l_{k})}$$

where L(·) is a weighting function that takes the global mean value $\mu_{k}$ of color image $B_{k}$ and the local mean value of current patch $b_{k}$ as inputs. L(·) quantifies the exposure of $b_{k}$ in $B_{k}$. It adopts a two-dimensional Gaussian profile to specify this measure as follows.

(12) $$L\left(\mu_{k},\sigma_{k}\right)=\exp\left(-\frac{{\left(\mu_{k}-\mu_{c}\right)}^{2}}{2\sigma_{g}^{2}}-\frac{{\left(l_{k}-l_{c}\right)}^{2}}{2\sigma_{l}^{2}}\right)$$

where $\sigma_{g}$ and $\sigma_{l}$ control the spreads of the profile along $\mu_{k}$ and $l_{k}$ dimensions, respectively. $\mu_{c}$ and $l_{c}$ as the constants of mid-intensity values are preset for calculation. For example, both $\mu_{c}$ and $l_{c}$ are 0.5 for the source image sequences normalized to [0, 1].

Once $\tilde{c}$, $\tilde{s}$, and $\tilde{l}$ are calculated, they uniquely define a new vector as follows.

(13) $$B_{k}^{p}=\tilde{c}\cdot \tilde{s}+\tilde{l}$$

The above operations are repeated for the base layer of all the source image sequences to achieve the optimization of image-block exposure quality, and the pixels in the overlapping image blocks are averaged to obtain an initial base layer of the fused image $B_{k}^{p}$. (more information of image patch structure decomposition can be found in [30].)

In the global exposure quality optimization of the base layer image, the Gaussian model is used to evaluate the exposure moderately. Considering the large-scale structure information of brightness, it uses a two-dimensional Gaussian function to evaluate the overall image exposure comprehensively. For each pixel at the (x,y) position in the base layer of each image, the blending weight is calculated by Equation (14).

(14) $$W_{k}^{B}\left(x,y\right)=\exp\left(-\frac{{\left(B_{k}\left(x,y\right)-0.5\right)}^{2}}{2\sigma_{l}^{2}}-\frac{{\left(\bar{L}-0.5\right)}^{2}}{2\sigma_{g}^{2}}\right)$$

The above two-dimensional Gaussian function is used to evaluate the global exposure quality of the base layer features, which is used in the final fusion. The global exposure optimization is realized by Equation (15). $B_{f}$ represents the weighted sum of the base layer products of each image in a set of input images and its corresponding weight in the fused image.

(15) $$B_{f}=\sum_{k=1}^{K}W_{k}^{B}B_{k}^{p}$$

#### 3.2.3. Exposure Fusion Using the Gaussian Weight Method

For the detail layer, the exposure features are calculated at each pixel position as the average luminance in a small local neighborhood, which is used to analyze the light and optimal dark changes of different pixels. Then, the values of each pixel in the detail layer under the optimal exposure mode are estimated based on the mean level of local intensity variations. The difference between each detail layer pixel value of the input image and the optimal pixel value is evaluated for the moderate exposure. The evaluation model of exposure moderation is shown in Equation (16). Accordingly, for each position $\left(x,y\right)$ in the detail layer of the $k^{\mathrm{th}}$ input image, the evaluation value $W_{k}^{D}\left(x,y\right)$ in exposure mode is obtained as follows.

(16) $$W_{k}^{D}\left(x,y\right)=\exp\left(-\frac{{\left(\varphi_{k}^{D}\left(x,y\right)-c_{e}\right)}^{2}}{2\sigma_{D}^{2}}\right)$$

where $\varphi_{k}^{D}$ denotes the exposure feature and is simply calculated by convolving the luminance component $L_{k}$ with a 7×7 average filter, σ controls the spread of Gaussian, and $c_{e}$ indicates the good exposure constant, which is normally set to the middle of the intensity range. The fused detail layer can be expressed by Equation (17).

(17) $$D_{f}=\sum_{k=1}^{K}W_{k}^{D}D_{k}$$

Once the fused base layer *B* and the detail layer *D* are calculated, the final multi-exposure fused image can be obtained by Equation (18).

(18) $$F=B_{f}+\alpha D_{f}$$

where α≥1 controls the detail strength and local contrast of the fused image *F*.

It tests the proposed method on several multi-exposure image sequences with different numbers of exposure levels. In the experiments, the parameters of the proposed method were set as follows. *d* that is inherited from the corresponding normalization term of SSIM equaled $\frac{1}{2}{\left(0.03L_{d}\right)}^{2}$, where $L_{d}$ is the maximum intensity value of source sequences. The exponential parameter *p* and two Gaussian spread parameters $\sigma_{g}$ and $\sigma_{l}$ in the algorithm were jointly determined by maximizing MEF-SSIM on five static source sequences using a grid search method. The values of *p*, $\sigma_{g}$, and $\sigma_{l}$ were set as p=4, $\sigma_{g}=0.2$, and $\sigma_{l}=0.5$, respectively. Two thresholds $T_{s}$ and $T_{m}$ were crucial for the proposed algorithm to work with dynamic scenes in the presence of camera and/or object motion. According to previous experiment results, $T_{s}=0.8$ and $T_{m}=0.1$ made a good balance among the reliably identified inconsistent motions in the exposures and a low rate of false positive detection. The patch size N=21 provided a good balance between performance and complexity. The stride of the moving window was determined by $D=[\frac{N}{10}]$ accordingly. *r* is the filter radius, which was set as r=12. α controls the detail strength, which was set as α=1.1. These parameter values were decided based on the fusion performance.

#### 3.2.4. The Workflow of The Proposed FPM Algorithm

The proposed FPM algorithm shown in Algorithm 1 detected the motion inconsistency and the brightness mapping to correct ghost regions first and then completed the registration of multi-exposure images in dynamic scenes. Next, the algorithm performed the spatial-domain image decomposition, completed the base and detail layer fusion, and integrated the image block structure decomposition into the appropriate evaluation of exposure. Finally, it achieved the fusion of multi-exposure images by the global and local exposure optimization.
**Algorithm 1** The proposed FPM algorithm.**Input:**  Source image sequences $I_{k},1\leq k\leq K$**Output:**  A fused image *F*1:Filter source images to select a reference image $I_{r}$2:Check the structure consistency K−1, and create latent images $\left\{{I^{\prime }}_{k}\right\}=\left\{{I^{\prime }}_{k}|k\neq r\right\}$ of $I_{r}$ by using IMF3:Replace the image block in $I_{k}^{\prime }$ with the image block in $I_{r}$ to obtain $M_{k}$4:**for** each image $M_{k}$ **do**5: Calculate $B_{k}$ and $D_{k}$ by using a guided filter6: **for** each $B_{k}$ **do**7:  Calculate $c_{k}$, $s_{k}$, and $l_{k}$ separately by Equations (6) and (7)8: **end for**9: Reconstruct the fused patch $B_{k}^{P}={\tilde{c}}_{k}\cdot {\tilde{s}}_{k}+{\tilde{l}}_{k}$10: Calculate the fusion weight of $B_{f}$ and $D_{f}$11:**end for**12:Combine the fused patches into *F* by Equation (14)

## 4. Comparative Experiments

### 4.1. Experiment Preparation

In the comparative experiments, multi-exposure source image sequences from both 24 sets of static scenes and 15 sets of dynamic scenes were processed. In total, 39 sets of multi-exposure source image sequences were processed by the adaptive patch structure based MEF (APS) [24], the dense scale invariant feature transform based MEF (DSIFT-EF) [28], the exposure fusion method based on high dynamic range (EFM) [6], the fast exposure fusion using exposedness function (Fast-expo) [18], the fast MEF with median filter and recursive filter (FMMR) [9], the structural patch decomposition based MED (SPD-MEF) [30], and the proposed FPM, respectively. The fused images were compared in both subjective and objective ways. The source image sequences of both static and dynamic scenes were collected by Ma [31], Hu [14], and Sen [15]. This section only selects four and three image groups from the 24 static scene and 15 dynamic scene image groups, respectively, for demonstration. All the experiments were programmed by MATLAB 2016b and run on an Intel I9 7900X @ 3.30 GHz desktop with 16.00 GB RAM.

### 4.2. Comparison of The Fused Images from Static Scenes

**Figure 3:**
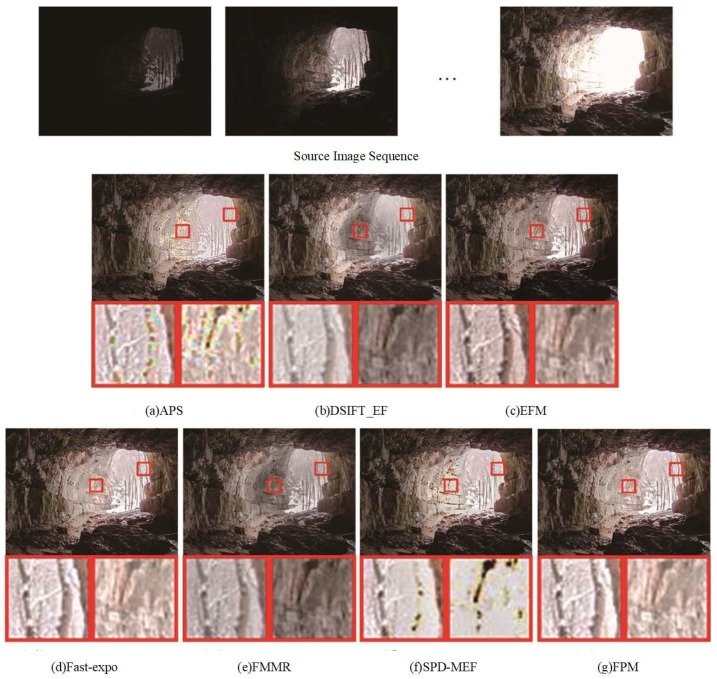
Comparison of the “Cave” fusion images obtained by the seven fusion methods. (**a**) APS, (**b**) DSIFT-EF, (**c**) EFM, (**d**) Fast-expo, (**e**) FMMR, (**f**) SPD-MEF, (**g**) FPM.

**Figure 4:**
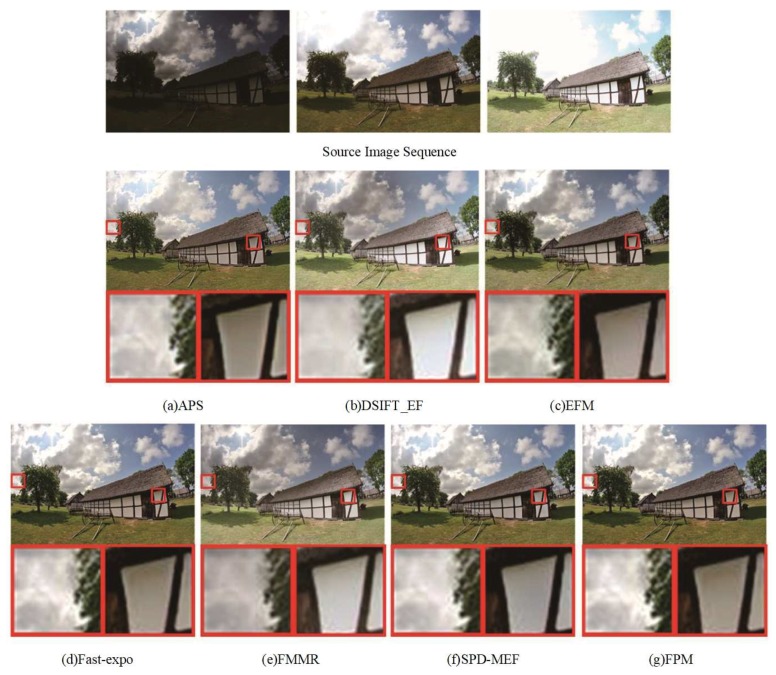
Comparison of the “Kluki” fusion images obtained by the seven fusion methods. (**a**) APS, (**b**) DSIFT-EF, (**c**) EFM, (**d**) Fast-expo, (**e**) FMMR, (**f**) SPD-MEF, (**g**) FPM.

**Figure 5:**
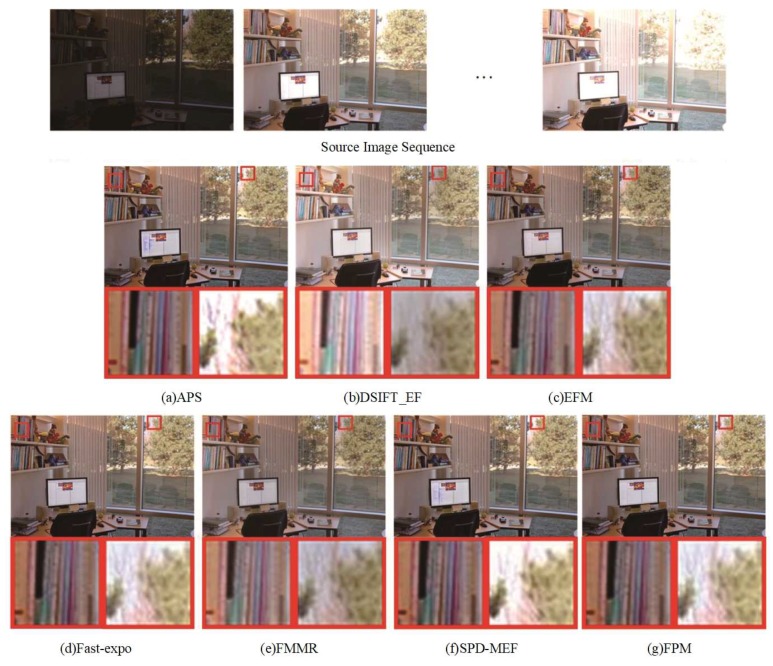
Comparison of the “Office” fusion images obtained by the seven fusion methods. (**a**) APS, (**b**) DSIFT-EF, (**c**) EFM, (**d**) Fast-expo, (**e**) FMMR, (**f**) SPD-MEF, (**g**) FPM.

Figure 3, Figure 4 and Figure 5 show the fused results of seven multi-exposure fusion methods in three different static scenes. The fused results of DSIFT-EF and FMMR had lower saturation and chromaticity than other methods. The color information of the stone wall in the center area of Figure 3b–e was lost. In Figure 4b–e, the texture details of the cloud in the partially enlarged area were lost. APS and SPD-MEF did not perform well in local brightness, sharpness, and gray-scale. In particular, the fusion results were partially overexposed and the pixels severely distorted in Figure 3a–f. In Figure 5b, the overall image brightness was high. As shown in the partially enlarged area of Figure 5b, the top of the tree had a low brightness, and the corresponding details were lost. Additionally, as shown in Figure 5a–f, the fused results of APS and SPD-MEF were excessively sharpened, in which the local colors were distorted and a blue color patch appeared. As shown in Figure 3c, the overall brightness of the fused result obtained by EFM was lower than the ones obtained by Fast-expo and FPM. However, the texture of ground rock area in the bottom right corner had a low contrast, and the structure detail information of the chair back was lost in Figure 5c. Overall, Fast-expo and FPM performed well in contrast, local saturation, and detail preservation. It was difficult to distinguish the difference between them by the human visual system.

### 4.3. Comparison of The Fused Images from Dynamic Scenes

**Figure 6:**
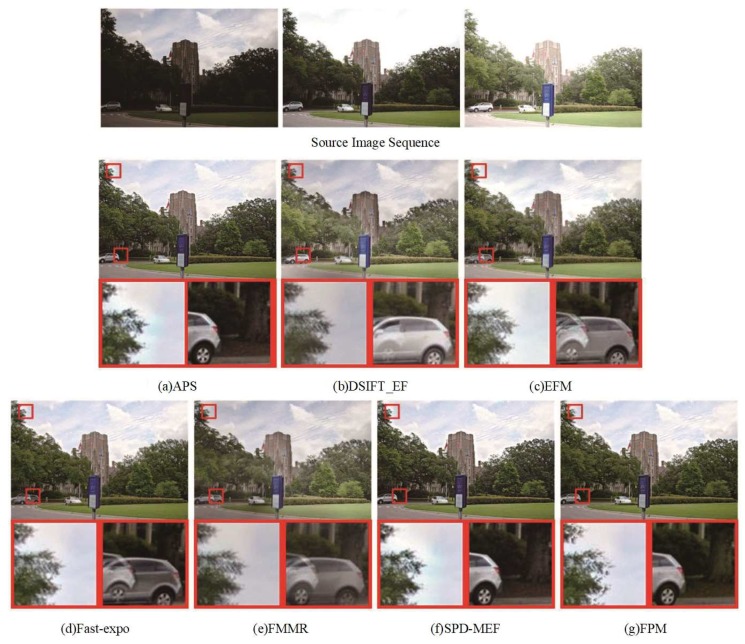
Comparison of the “Duke” fusion images obtained by the seven fusion methods. (**a**) APS, (**b**) DSIFT-EF, (**c**) EFM, (**d**) Fast-expo, (**e**) FMMR, (**f**) SPD-MEF, (**g**) FPM.

**Figure 7:**
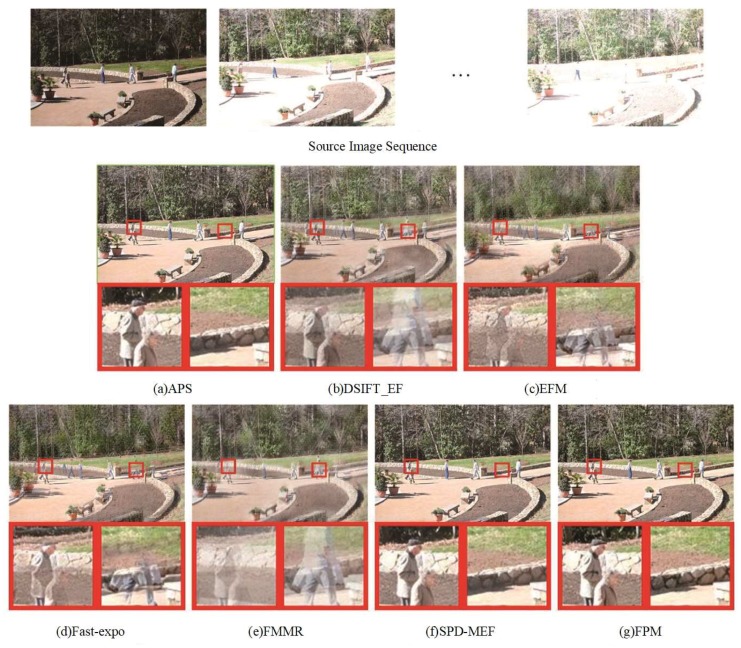
Comparison of the “Garden” fusion images obtained by the seven fusion methods. (**a**) APS, (**b**) DSIFT-EF, (**c**) EFM, (**d**) Fast-expo, (**e**) FMMR, (**f**) SPD-MEF, (**g**) FPM.

As shown in Figure 6 and Figure 7, the DSIFT-EF, EFM, Fast-expo, and FMMR methods lacked the motion estimation, and the fused results contained ghosts. APS and SPD-MEF could effectively remove the ghosts in the fused images. Due to the lack of the a priori exposure quality information from the reference image, the connected area between tree and sky had a serious dispersion phenomenon as shown in the partially enlarged areas of Figure 6a,f, compared to the corresponding result of the proposed FPM. The MEF results shown in Figure 6 and Figure 7 confirmed the performance of reference image exposure quality in dynamic scenes. The usage of a priori information could effectively remove the ghosts, reduce the influence of overexposed areas from the reference image in the fused results (such as dispersion, noise), and improve the visual quality with respect to human eyes.

Combining the fused results of both dynamic and static scenes, APS and SPD-MEF could effectively remove the ghost areas, but performed poorly in the local brightness, sharpness, and gray scale.

Fast-expo could achieve a precise fusion of multi-exposure images in static scenes, but could not effectively remove the ghosts in dynamic scenes. In general, compared with the other six methods, the proposed FPM could effectively remove the ghosts in dynamic scenes, and the fused results in static scenes had better performance for the human visual system.

### 4.4. Objective Evaluation Index

**Figure 8:**
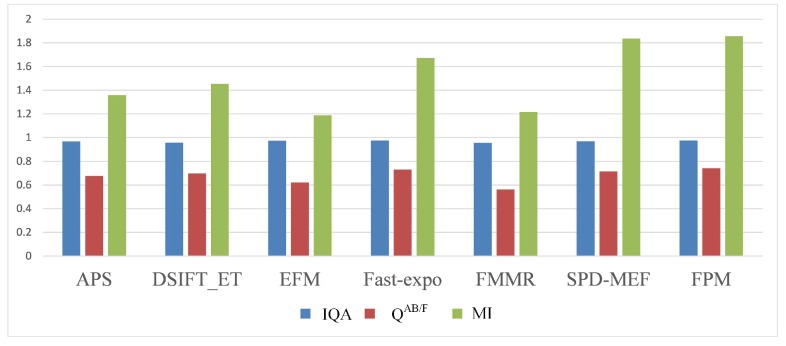
Mean value histogram of objective evaluation indicators obtained by the seven fusion methods.

Three objective evaluation indicators were used to evaluate the fusion performance objectively. The quality perception indicator IQA proposed by Ma was used to evaluate the structure consistency between the fused result and source image sequence [37]. As gradient based quality indices, both $Q^{AB/F}$ [38,39] and MI [20,40] were used to measure the edge information and the similarity between the fused image and source images. The average IQA, $Q^{AB/F}$, and MI of the total of 39 sets of fused images obtained by the seven different ways are shown in Figure 8 and Table 1. In Table 1, the best results of three objective evaluation indicators are marked in bold.

The value range of IQA was between zero and one. When the value increased, the fused result had a better structure consistency with the source image sequences. As shown in the fused results obtained by DFIFT-ET and FMMR, the structure of each fused result had a low similarity to source image sequences, and the preservation capability of the detail information was insufficient, so it caused the reduction of both saturation and chromaticity. Meanwhile, the other five methods had better corresponding performances. The average objective evaluation indicators of EFM, Fast-expo, and the proposed FPM were the top three among all seven methods. They all had excellent performances in structure similarity. The proposed FPM obtained 0.9746 in IQA, which was a little bit better than EFM’s 0.9733 and Fast-expo’s 0.9744.

Similarly, for both $Q^{AB/F}$ and MI, the larger value meant the better performance in the preservation of edge information and the similarity between the fused image and source images. The proposed FPM also achieved the best performance in both $Q^{AB/F}$ and MI. Comparing with EFM and Fast-expo, the proposed FPM had significantly better performances. For $Q^{AB/F}$, FPM obtained 0.7411, but EFM and Fast-expo only obtained 0.6214 and 0.7301, respectively. Similarly, FPM obtained 1.8551 in MI, but EFM and Fast-expo only obtained 1.1876 and 1.6724, respectively. The corresponding $Q^{AB/F}$ and MI results are reflected in Figure 6 and Figure 7. The proposed FPM had better performance than EFM and Fast-expo in dynamic scenes. As the second best result in MI, the performance of SPD-MEF was close to the proposed FPM in MI, and its performances in IQA and $Q^{AB/F}$ were obviously worse than the proposed FPM.

Overall, the proposed FPM outperformed the other six methods in the preservation of structure and texture details, as well as the observation of the human visual system. It also achieved better performance in color saturation, sharpness, and local detail processing. In addition, compared with the other six methods, the proposed FPM could effectively remove the ghosts in the MEF of dynamic scenes. In summary, the proposed FPM achieved the best overall performance.

## 5. Conclusion and Future Work

This paper proposed a novel multi-exposure image registration and fusion method based on the a priori exposure and low-level features (FPM) to achieve the precise fusion of both dynamic and static scenes. It achieved the image registration through the a priori exposure quality and structure consistency checks to reduce the ghosts in the dynamic scene fusion. Spatial-domain decomposition, image patch structure decomposition, and the appropriate exposure evaluation were used to achieve the accurate fusion of multi-exposure images.

The fusion performance of the proposed algorithm was tested by using different multi-exposure images from different scenes. In static scenes, this method could preserve more image details and perform well in color saturation, sharpness, and local detail processing. In dynamic scenes, the ghosts could be effectively removed to improve the visual quality of the fused result. Therefore, the proposed algorithm achieved the precise fusion of both static and dynamic scenes.

Although the proposed fusion method could effectively remove the ghosts and achieve the precise fusion, the computation efficiency was relatively low. The high computation cost of the proposed method may be caused by the following reasons, such as an image subjected to the a priori estimation of exposure quality, the connectivity and structure consistency detection in the ghost removal, and the global and local exposure quality optimization in the fusion of base layers. These processes calculated and counted the pixel/patch features of all the source images, which increased the computation costs. The ghost detection and removal spent about 60% of the total computation costs. Therefore, in the future, a more concise motion-consistency detection algorithm will be designed to reduce the computation costs of ghost detection in dynamic scenes. Additionally, in the static scene image fusion, the parameters of the fusion algorithm will be simplified, and the algorithm calculation steps will also be optimized to improve the fusion efficiency.

## Figures and Tables

**Table 1:** Mean value of the objective evaluation indicators obtained by the seven fusion methods.

	APS	DSIFT-EF	EFM	Fast-expo	FMMR	SPD-MEF	FPM
IQA	0.9678	0.9573	0.9733	0.9744	0.9557	0.9693	**0.9746**
$Q^{AB/F}$	0.6765	0.6973	0.6214	0.7301	0.5623	0.7147	**0.7411**
MI	1.3598	1.4552	1.1876	1.6724	1.2157	1.8353	**1.8551**
